# Genetically predicted smoking and body mass index mediate the relationship between insomnia and myocardial infarction

**DOI:** 10.3389/fcvm.2024.1456918

**Published:** 2024-11-13

**Authors:** Limei Deng, Yuan Gao, Dongmei Wan, Zheng Dong, Yuming Shao, Jing Gao, Wenji Zhai, Qian Xu

**Affiliations:** ^1^ The Second Affiliated Hospital of Heilongjiang University of Chinese Medicine, Harbin, Heilongjiang, China; ^2^ Heilongjiang University of Chinese Medicine, Harbin, Heilongjiang, China

**Keywords:** insomnia, myocardial infarction, smoking, body mass index, alcohol consumption, Mendelian randomization

## Abstract

**Objective:**

This study aimed to investigate the causal relationship between insomnia and the risk of myocardial infarction (MI) and explore potential mediators such as smoking initiation, alcohol consumption and body mass index (BMI) using mendelian randomization (MR) analysis.

**Methods:**

Data from 1,207,228 individuals of European ancestry were obtained from the UK Biobank and 23andMe for insomnia-related genetic associations. Genetic instruments for MI, smoking initiation, alcohol consumption, and BMI were derived from large-scale genome-wide association studies. Univariate MR analysis mainly utilized the inverse variance weighting method, and multivariable MR analysis assessed the mediation effects of smoking initiation and BMI.

**Results:**

The univariate MR analysis revealed a 96% increased risk of MI in individuals with insomnia [odds ratio (OR) = 1.96; 95% CI: 1.67, 2.31]. Smoking initiation and BMI were identified as potential mediators. The multivariable MR analysis indicated smoking initiation accounted for 29% of the total effect (95% CI: 13%, 61%), while BMI accounted for 15% (95% CI: 7%, 27%), with a combined mediation proportion of 54% (95% CI: 31%, 91%).

**Conclusions:**

The results of this MR analysis demonstrate that insomnia increases the risk of MI. Quitting smoking and losing weight may reduce this risk; however, there is still a portion of the impact of insomnia on MI that cannot be explained. Therefore, further investigation into other potentially modifiable intermediate factors is necessary.

## 1 Introduction

Myocardial infarction (MI) is a serious cardiovascular disease, typically caused by the blockage of coronary arteries, leading to myocardial ischemia and necrosis (1). With the changes in social lifestyles and the increase in unhealthy habits, the incidence of MI is gradually rising, exerting a significant impact on public health, prompting scientists to delve into its potential influencing factors (2). Epidemiological studies have revealed high-risk factors for MI, including hypertension (3), hyperlipidemia (4), diabetes (5) and so on. In recent years, research on the relationship between insomnia and MI has gradually attracted widespread attention in the academic community.

Insomnia is the most common sleep disorder, known to have a negative impact on the overall health and quality of life of the population (6). Recent research reports indicated a high prevalence of insomnia in the United States, ranging from 10% to 15% (7). An increasing number of studies suggested a close association between insomnia and MI (8, 9), but the limitations of observational studies cannot be ignored. Additionally, lifestyle factors such as smoking (10, 11), alcohol consumption (12, 13), and body mass index (BMI) (14, 15) have also been confirmed to be associated with insomnia. However, these relationships are often limited by observational studies, requiring more in-depth research methods to reveal potential causal effects.

Mendelian randomization (MR) analysis provides a powerful tool in addressing the limitations of observational studies and revealing causal effects. By using genetic variations as instrumental variables (IVs) to assess the causal impact of exposure on outcomes, it effectively mitigates the potential influence of confounding factors, enhancing the reliability of study results (16, 17). This study aims to use MR methods to delve into whether smoking, alcohol consumption, and BMI mediate the causal effects of insomnia on MI, providing a scientific basis for developing more effective cardiovascular health interventions. Through this research, we hope to gain a more comprehensive understanding of the intricate relationship between lifestyle and cardiac health, offering more precise guidance for the prevention and treatment of MI.

## 2 Materials and methods

### 2.1 Data sources

The study exclusively involved individuals of European ancestry as other ancestral groups were not universally available for all features of interest, and due to genetic population structure, the mixing of ancestries could introduce confounding. Genetic associations related to insomnia come from the UK Biobank and 23andMe (18), with this analysis incorporating a large sample of 1,207,228 individuals of European ancestry, making it one of the largest genome-wide association study (GWAS) studies on insomnia to date. To explore the genetic associations with MI, we acquired data from another GWAS analysis, encompassing 395,795 participants and 10,290,368 SNPs (19). Genetic instruments for smoking initiation and alcohol consumption are derived from statistical data in a large-scale publicly available genome-wide association meta-analysis (20). In this study, a standardized analysis was conducted across 34 individual cohorts, considering adjustments for age, age squared, gender, and genetic ancestry principal components. The effects sizes of individual single nucleotide polymorphisms (SNPs) on smoking and alcohol consumption, along with their respective standard errors, were assessed. In addition, data related to BMI came from a GWAS involving 806,834 individuals of European ancestry (21). BMI values were obtained through measurements of standing height and weight during the initial assessment center visit. Table 1 presented a summary of the studied GWAS information.

**Table 1:** Summarized information for GWASs included.

Study	Authors	PMID	Sample size	No. of SNPs	R^2^	F
Insomnia	Watanabe et al.	35835914	1,207,228	429	1.65E-05	19.87
Smoking initiation	Liu et al.	30643251	1,232,091	77	1.49E-05	18.33
Alcohol consumption	Liu et al.	30643251	941,280	34	2.48E-05	23.38
BMI	Pulit et al.	30239722	806,834	60	3.03E-05	24.48
Myocardial infarction	Hartiala et al.	33532862	395,795	NA	NA	NA

GWAS, Genome-wide association studies, BMI, body mass index; SNP: single nucleotide polymorphism.

### 2.2 Genetic instrument selection

In our MR analysis, we utilized SNPs as IVs to estimate the overall impact of insomnia on MI. When the genome-wide significance threshold was *P* < 5 × 10^−8^, SNPs associated with each exposure were selected as potential IVs. To ensure the independence of genetic variations used as IVs, we set the linkage disequilibrium threshold for grouping to R^2^ < 0.01, with a window size of 1,000 kb. If suitable replacement SNPs were not available, the SNP was discarded. Simultaneously, we set a minimum allele frequency (MAF) of 0.3 to ensure SNP commonality (22, 23). Additionally, we standardized genetic variations from various studies based on their effects, excluding any palindromic variations. To assess the strength of the instruments, we calculated the F-statistic. We consider an F-statistic greater than 10 as an indicator of robust instrument strength. The F-statistic was calculated using the formula ((N-K-1)/K × [R^2^/(1−R^2^)]), where R^2^ was the variance explained by each SNP for each exposure, N was the sample size of the exposure GWAS, K was the number of SNPs, and the R^2^ formula was expressed as R^2^ = 2 × MAF × (1 − MAF) × [*β*^2^/(SE^2^ × N)], where MAF represented the minor allele frequency, *β* was the effect size of the exposure, and SE denoted the standard error of the effect size (24).

### 2.3 Statistical analysis

#### 2.3.1 Univariate MR analysis

In our study, we employed the Inverse Variance Weighting (IVW) method as the primary approach for univariate analysis. This technique utilized a random-effects meta-analysis approach to combine results obtained from individual SNPs. To ensure consistency in the causal direction, we also conducted additional analyses using other methods: MR Egger, Weighted Median, Weighted Mode, and Simple Mode. To ensure the reliability of the results, significance in the IVW method results was required, and the results from the other four methods should be directionally consistent with the IVW results. To control for the type I error rate, the Benjamini–Hochberg method was used to adjust for multiple testing. The false discovery rate (FDR) threshold was set at 0.05 for significance. After the MR analysis, Cochran's Q test was used to assess the heterogeneity of the ratio estimates of the IVs related to the exposure on the outcome risk (25). When test results indicated heterogeneity (*P* < 0.05), we introduced the MR-PRESSO method to remove IVs with heterogeneity and then reanalyzed those not identified as heterogeneous IVs (26). We also employed the MR-Egger intercept method to assess evidence of horizontal pleiotropy within selected SNPs (the presence of horizontal pleiotropy was speculated for *P* < 0.05).

#### 2.3.2 Multivariable MR analysis

**Figure 1:**
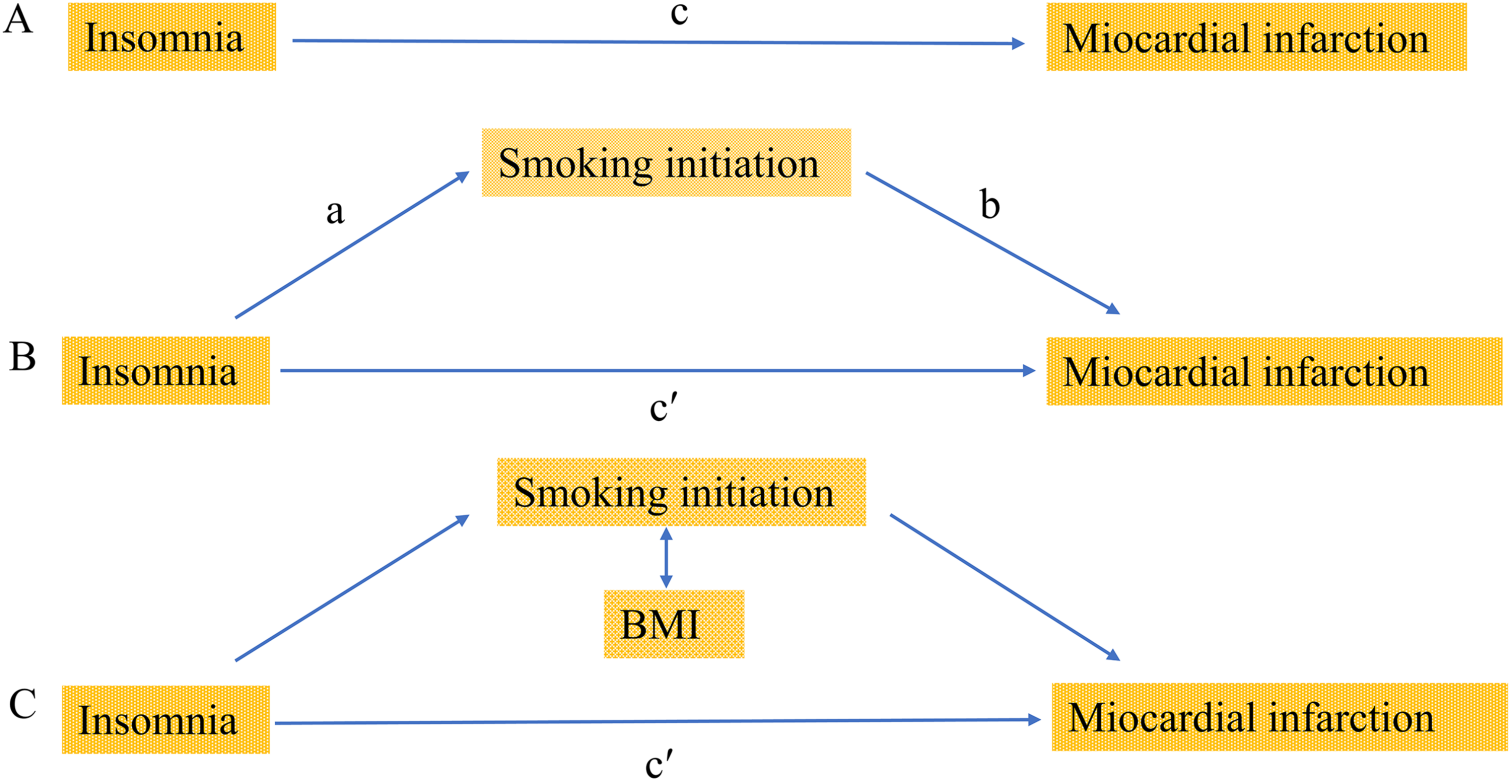
Diagrams illustrating associations examined in this study. **(A)** The total effect of insomnia on miocardial infarction (MI), c, was derived using univariable MR (i.e. genetically predicted insomnia as exposure and miocardial infarction as outcome). **(B)** The total effect was decomposed into: (i) indirect effect using a two-step approach (where a is the total effect of insomnia on smoking initiation, and b is the effect of smoking initiation on MI) and the product method (a × b) and (ii) direct effect (c′ = c − a × b). The same process applied to mediation analysis of body mass index (BMI). **(C)** For mediation by both smoking initiation and BMI combined (arrows represent their bidirectional causal relationship), the indirect effect was derived using the difference method (c − c′). Proportion mediated was the indirect effect divided by the total effect.

In our study, we utilized multivariable MR to investigate the causal relationship between insomnia and MI risk, with smoking initiation and BMI as mediators. The specific methods were follows: firstly, the estimated value of the mediating effect of insomnia (a in Figure 1B) was multiplied by the estimated value of the mediating effect on MI (b in Figure 1B) to obtain the estimate of the indirect effect. Then, the indirect effect was divided by the total causal effect of insomnia on MI (c in Figure 1A) to obtain the proportion of mediation. In the presence of multiple mediators, the difference method was employed, subtracting the direct effect from the total causal effect (c’ in Figure 1C) to obtain the indirect effect. Then, we further divide the indirect effect by the total causal effect to determine the proportion of mediated mediation.

All statistical analyses in this study were conducted using the “TwoSampleMR” package in R software (version 4.1.0). All presented *P*-values were two-sided, and statistical significance was set at the 5% level. We adhered to the guidance of the STROBE-MR guidelines when reporting MR studies (27).

## 3 Results

### 3.1 Instrumental variables

Summary data of SNP-phenotype associations were obtained from GWAS for each phenotype (Supplementary Tables S1–S4). A total of 429 SNPs were chosen as IVs for insomnia, with an F-statistic of 19.87. For smoking initiation, alcohol consumption, and BMI, we extracted 77 SNPs (F = 18.33), 34 SNPs (F = 23.38), and 60 SNPs (F = 24.48), respectively, ensuring the absence of weak IVs.

### 3.2 Effects of insomnia and potential mediators on MI

**Figure 2:**
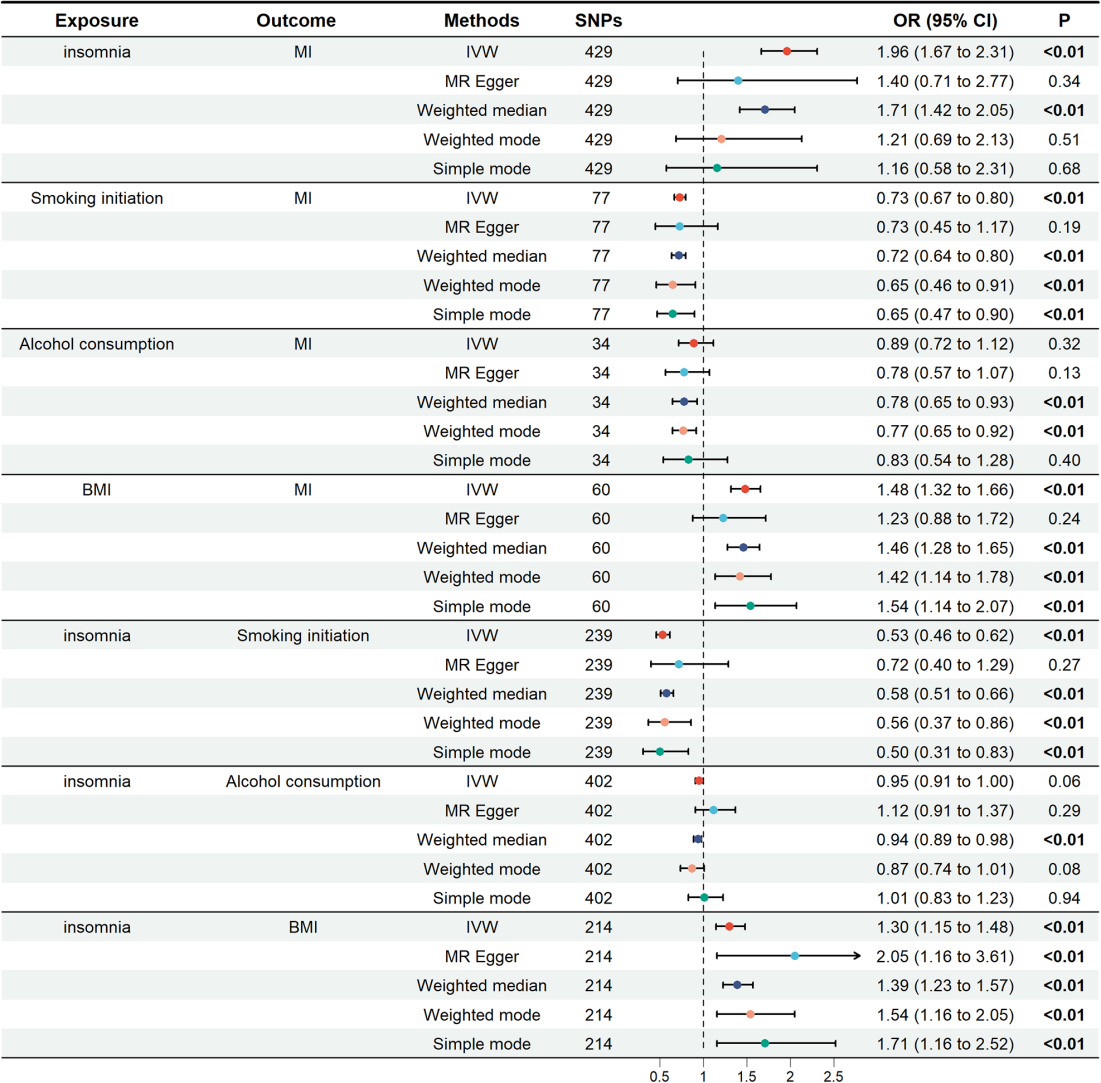
Univariate Mendelian randomization analysis. The figure showed the impact of insomnia and potential mediators on MI, as well as the effects of insomnia on potential mediators. Potential mediators include smoking initiation, alcohol consumption and BMI. MI, miocardial infarction; BMI, body mass index; SNPs, single nucleotide polymorphisms; OR, odds ratio; IVW, inverse variance weighting.

In the univariate mendelian randomization (UVMR) analysis, the risk of MI increased by 96% in patients with insomnia, with an odds ratio (OR) of 1.96 (95% CI: 1.67, 2.31). For the three potential mediators analyzed, alcohol consumption was not associated with MI (OR = 0.89; 95% CI: 0.72, 1.12). The unadjusted associations of smoking initiation and BMI with MI were 0.73 (95% CI: 0.67–0.80) and 1.48 (95% CI: 1.32–1.66), respectively. The impact of insomnia on smoking initiation was (OR = 0.53; 95% CI: 0.46, 0.62), as well as on alcohol consumption (OR = 0.95; 95% CI: 0.91, 1.00) and BMI (OR = 1.30; 95% CI: 1.15, 1.48). These findings were depicted in Figure 2.

### 3.3 Mediation analysis

**Figure 3:**
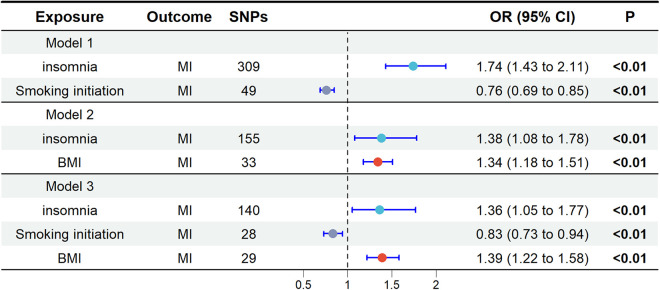
Multivariate Mendelian randomization analysis. The OR is derived from the method of inverse variance weighting. BMI, body mass index; MI, miocardial infarction; OR, odds ratio; SNPs, single nucleotide polymorphisms.

In the UVMR analysis, alcohol consumption had no effect on MI and was therefore excluded from subsequent multivariable mendelian randomization (MVMR) analysis. When screening potential mediators, we constructed three models, each adjusting for different mediators, as shown in Figure 3. Consistent results indicated a direct impact of insomnia on MI, with no evidence of complete mediation. In Model 1, the adjusted causal effect of smoking initiation on MI was OR = 0.76 (95% CI: 0.69, 0.85). In Model 2, the adjusted causal effect of BMI on MI was OR = 1.34 (95% CI: 1.18, 1.51). In Model 3, the adjusted causal effects of smoking initiation and BMI on MI were OR = 0.83 (95% CI: 0.73, 0.94) and OR = 1.39 (95% CI: 1.22, 1.58), respectively.

In the MR analysis, the total effect of insomnia on MI was 0.674 (95% CI: 0.512, 0.836). The direct effect in the MR analysis was 0.309 (95% CI: 0.045, 0.573). Mediation analysis showed that in the MR study, smoking initiation accounted for 29% of the total effect (95% CI: 13%, 61%), while BMI accounted for 15% (95% CI: 7%, 27%), with a combined mediation proportion of 54% (95% CI: 31%, 91%) see Table 2 for details.

**Table 2:** Estimate of the effect of insomnia on MI explained by each mediator and by both combined.

Methods	Exposures	Outcome	*β* (95% CI)	*p*-value
UVMR	Insomnia	MI	0.674（0.512, 0.836）	<0.001
	Insomnia	Smoking initiation	−0.626（−0.771, −0.481）	<0.001
	Insomnia	BMI	0.264（0.137, 0.390）	<0.001
	Smoking initiation	MI	−0.317 (−0.405, −0.229)	<0.001
	BMI	MI	0.391 (0.276, 0.506)	<0.001
MVMR	Insomnia	MI	0.309 (0.045, 0.573)	0.022

UVMR, univariate mendelian randomization; MVMR, multivariable mendelian randomization; MI, myocardial infarction; BMI, body mass index.

### 3.4 Sensitivity analysis

In the MR analysis, heterogeneity tests revealed significant heterogeneity among the selected IVs (*P* < 0.05). Given this, the IVW random-effects model was used in all MR analyses (Table 3). MR-Egger_intercept analyses did not detect potential horizontal pleiotropy (*P* > 0.05), indicating that IVs did not significantly affect the outcome through pathways outside the exposure (Table 4).

**Table 3:** Heterogeneity tests for UVMR analysis.

Exposure	Outcome	Methods	Q	Q-df	Q-pval
Insomnia	MI	MR Egger	892.61	427	4.56E-35
Insomnia	MI	IVW	894.69	428	3.83E-35
Smoking initiation	MI	MR Egger	112.51	75	3.30E-03
Smoking initiation	MI	IVW	112.51	76	4.15E-03
Alcohol consumption	MI	MR Egger	91.29	32	1.31E-07
Alcohol consumption	MI	IVW	95.39	33	5.65E-08
BMI	MI	MR Egger	125.78	58	6.45E-07
BMI	MI	IVW	128.62	59	4.35E-07
Insomnia	Smoking initiation	MR Egger	987.44	237	3.52E-92
Insomnia	Smoking initiation	IVW	991.69	238	1.43E-92
Insomnia	Alcohol consumption	MR Egger	1,114.66	400	1.06E-68
Insomnia	Alcohol consumption	IVW	1,121.40	401	2.03E-69
Insomnia	BMI	MR Egger	552.51	212	3.42E-32
Insomnia	BMI	IVW	559.31	213	6.66E-33

UVMR, univariate Mendelian randomization; MI, myocardial infarction; BMI, body mass index; IVW, inverse variance weighting. Q, Cochran's Q statistic in IVW and MR-Egger; df, (number of) degrees of freedom; Q-pval, the null hypothesis (H0) for Q-pval is that there is no difference between each SNP.

**Table 4:** Horizontal pleiotropy test for UVMR analysis.

Exposure	Outcome	Egger-intercept	SE	*p*-value
Insomnia	MI	0.002	0.002	0.319
Smoking initiation	MI	<0.001	0.006	1.000
Alcohol consumption	MI	0.004	0.004	0.239
BMI	MI	0.005	0.005	0.257
Insomnia	Smoking initiation	−0.002	0.002	0.314
Insomnia	Alcohol consumption	−0.001	0.001	0.121
Insomnia	BMI	−0.003	0.002	0.108

UVMR, univariate Mendelian randomization; MI, myocardial infarction; BMI, body mass index. Egger-intercept represents the intercept computed using the MR-Egger method. The *p*-value associated with Egger-intercept refers to the hypothesis test conducted on the intercept, where the null hypothesis is that the intercept is equal to zero.

## 4 Discussion

Our study results support a potential causal relationship between genetically predicted insomnia and MI risk. Compared to the general population, insomnia patients have a 96% increased risk of MI. Of this impact, 29% is mediated by smoking, and 15% is mediated by BMI. These two major risk factors for MI account for 54% of the total effect of insomnia. To our knowledge, this is the first application of MR mediation analysis to study mediators of insomnia and MI risk.

Our study results were consistent with previous study on the impact of insomnia on MI. Hu et al.'s meta-analysis found that in individuals with no prior MI, insomnia was associated with a higher risk of MI (9). Similar results were also found in another meta-analysis (28). The study found that patients with insomnia, compared to healthy individuals, experience changes in the hypothalamic-pituitary-adrenal axis, with a significant increase in adrenocortical hormones and cortisol levels (29). Elevated cortisol was associated with MI, as evidenced by a significant increase in cortisol levels in patients with acute MI one month before the onset of the disease (30). Animal experiments suggest that chronic stress and elevated cortisol can accelerate atherosclerosis, potentially leading to MI (31). Insomnia patients, due to insufficient sleep, suffer from chronic stress and elevated cortisol, exacerbating their risk of MI.

The mediation analysis results emphasize that in the causal relationship between insomnia and MI, smoking and BMI serve as key intermediate variables. This finding was consistent with past research results, highlighting the close association between insomnia and higher rates of smoking and obesity, both identified as factors increasing the risk of MI (15, 32, 33). Smoking, as a behavioral habit, was not only closely associated with insomnia but has also been confirmed as an independent risk factor for MI (34). This suggests that insomnia may increase the risk of MI by promoting smoking behavior. Meanwhile, BMI, as an indicator of physical health, plays a crucial mediating role between insomnia and MI. There is an association between obesity and insomnia, and higher BMI has been confirmed to be closely related to heart disease (35). Therefore, insomnia may trigger complex and interwoven effects in the pathogenesis of MI by influencing smoking and BMI. The results of this study provide valuable insights for the management and prevention of heart disease. When formulating intervention strategies, it is essential to not only focus on insomnia itself but also comprehensively consider the associated behaviors and physiological factors, especially smoking and BMI. Taking these intermediate variables into account may contribute to the development of more effective measures to reduce MI risk.

Another interesting aspect of our study was the finding that smoking and BMI jointly mediate 54% of the impact of insomnia on MI, while there was still a portion of the impact that remains unexplained. It is well known that insomnia may trigger depression and emotional fluctuations, and depression itself is considered an independent risk factor for MI (36, 37). Under depressive states, biological changes may lead to an imbalance in hormone levels, such as increased release of cortisol and overactivity of the autonomic nervous system (38, 39). These physiological changes are closely related to the health of the cardiovascular system, thereby increasing the risk of MI. Furthermore, factors such as the triglyceride glucose index-waist circumference (40), hepatic steatosis index (41), plasma aldosterone concentration (42), and weight-adjusted-waist index (43) may also serve as potential risk factors for MI. These indices reflect metabolic and hormonal disturbances that can exacerbate cardiovascular issues, particularly in individuals experiencing insomnia and depression. Additionally, depression may lead to unhealthy lifestyle choices, such as lack of exercise and poor dietary habits, further aggravating heart health issues. Therefore, insomnia indirectly influences the risk of MI by triggering depression, highlighting the intricate relationship between sleep, mental health, biology, and heart disease. Consequently, these non-lifestyle factors deserve further investigation as mediators of the impact of insomnia on MI.

This study has several notable strengths. Firstly, despite previous MR studies on insomnia and MI (44, 45), we are the first to use the mediation MR method to study the causal relationship between insomnia and MI and discover the mediating roles of smoking and BMI. Secondly, we utilized summary-level data from recent extensive sample studies in GWAS, covering a more comprehensive set of SNPs, thereby enhancing the statistical power of the data. Finally, employing the mediation MR method helped reduce biases caused by confounding between exposure factors, mediators, outcomes, and measurement errors.

However, we should not overlook several limitations. Firstly, although smoking and BMI largely explain the impact of insomnia on the risk of MI, the remaining portion of the effect still needs further exploration. Secondly, despite some heterogeneity in this study, our data came from public databases, and specific factor sub-analyses (such as age, gender, etc.) to explore the source of heterogeneity were not feasible. Additionally, the overlap in samples from the UK Biobank for both insomnia and MI may also impact our findings, but quantifying this overlap was not possible due to data source limitations. Future research on the correlation between insomnia and MI should consider the overall impact of these factors. Finally, GWAS summary data primarily came from European populations, so there may be some potential biases if extrapolated to non-European ancestries. These limitations emphasize the need for further research to refine the evidence in this field.

## 5 Conclusions

In summary, we demonstrated the hazardous effect of insomnia on the risk of MI. Quitting smoking and losing weight may help reduce this risk, especially crucial for those at higher risk of MI. However, nearly half of the impact of insomnia on MI remains difficult to explain. To comprehensively reduce the risk, efforts to improve lifestyle habits need to be strengthened, and further study is required to investigate other potential influencing factors not controlled by lifestyle factors.

## Data Availability

The datasets presented in this study can be found in online repositories. The names of the repository/repositories and accession number(s) can be found in the article/Supplementary Material.
